# Emergency ultrasound of respiratory muscles: a promising tool for determining the outcomes of COPD exacerbations

**DOI:** 10.55730/1300-0144.5664

**Published:** 2023-05-25

**Authors:** Dinçer YEGİN, Alten OSKAY, Tülay OSKAY, Selcan ENVER DİNÇ, Hande ŞENOL, Mert ÖZEN, Vefa ÇAKMAK, Murat SEYİT, Atakan YILMAZ, İbrahim TÜRKÇÜER

**Affiliations:** 1 Department of Emergency Medicine, Ardahan State Hospital, Ardahan, Turkiye; 2 Department of Emergency Medicine, Faculty of Medicine, Pamukkale University, Denizli, Turkiye; 3 Department of Cardiology, Bucak State Hospital, Burdur, Turkiye; 4 Department of Emergency Medicine, Kepez State Hospital, Antalya, Turkiye; 5 Department of Biostatistics, Faculty of Medicine, Pamukkale University, Denizli, Turkiye; 6 Department of Radiology, Faculty of Medicine, Pamukkale University, Denizli, Turkiye

**Keywords:** COPD, diaphragmatic thickness, diaphragmatic ultrasound, emergency department, intercostal muscle ultrasound, mortality

## Abstract

**Background/aim:**

The respiratory muscles of patients with chronic obstructive pulmonary disease (COPD) exhibit structural and functional changes that can be evaluated and monitored by ultrasonography.

**Materials and methods:**

This single-center, prospective study was conducted in the emergency department (ED) of a tertiary care hospital over an eight-month period (September 2020–May 2021). Diaphragmatic excursions, end-expiratory thickness, and thickening fractions, as well as right and left intercostal muscle thicknesses, of all adult subjects manifesting COPD exacerbation, were assessed. The data were analyzed regarding ward/intensive care unit (ICU) hospitalization or discharge from the ED, mortality, and readmission within 15 days.

**Results:**

Sixty-three subjects were recruited for the study. Diaphragmatic excursion, end-expiratory diaphragmatic thickness, and intercostal muscle thickness measurements were significantly different between the ward, ICU, and discharge groups (p < 0.001) but lower in the deceased subjects (all p < 0.05). The diaphragmatic excursion value of 3.25 cm was the threshold value measured for distinguishing discharge from ED, and 1.82 cm was measured for admission to the ICU, both with 100% sensitivity and selectivity (AUC = 1).

**Conclusion:**

Diaphragmatic excursion, diaphragmatic end-expiratory thickness, and right and left intercostal muscle thicknesses vary in the prognosis of subjects presenting with COPD exacerbation.

## 1. Introduction

Chronic obstructive pulmonary disease (COPD) is a major contributor to mortality and morbidity, most notably in developing countries^1^ [1,2]. COPD exacerbation accounts for a substantial share of emergency department (ED) admissions due to the increasing frequency in the condition, especially during the winter months [3]. Age, smoking, male gender, and comorbidities are key to both the occurrence of COPD and related mortality and morbidity^1^ [4].

Inflammation in the small airways, fibrosis, increased oxidative stress, protease-antiprotease imbalance, cellular aging and age-related changes, autoimmunity, infections, impaired immune regulation, and repair mechanisms might be the underlying factors responsible for the development and progression of COPD^1^ [5]. These also affect the structure of the respiratory muscles in patients with COPD [6]. Moreover, irreversible airflow limitation, as well as static and dynamic hyperinflation, are likely to occur as a result of the restructuring of the small airways and emphysematous destruction of the parenchyma [7]. With the resulting air trapping, the patient’s next breath volume is restricted, and the vicious circle gradually increases. Diaphragmatic structure, size, functions, and respiratory mechanics are also affected by this situation [6–9].

Respiratory muscles consist of type I and type II muscle fibers. Of these, type I fibers are slow-twitching and long-term contracting fibers without getting exhausted. They use an oxidative system to provide energy. Type II fibers, by contrast, provide energy through oxidative and glycolytic pathways. These are fast-twitching fibers that become exhausted more quickly [10]. The structure and strength of muscle fibers show changes with age, nutritional status, exposure to cigarette smoke, hypoxia, oxidative stress, the presence of COPD, and the use of certain medications [6,10]. In particular, the systemic and pulmonary effects of inflammation and oxidative stress in patients with COPD are well-documented [6,11,12]. In this regard, imaging with ultrasound is one of the methods used to evaluate and monitor impaired functions. There is accumulating empirical evidence for the need to monitor patients with COPD, evaluate their response to rehabilitation, decide on extubation, and assess the success of noninvasive mechanical ventilation with diaphragmatic ultrasound [13–17].

Within this context, our study sets out to assess the ultrasonographic features of the respiratory muscles and their relationship with the outcomes (hospitalization or discharge status, readmission, and mortality in 15 days) in subjects with acute COPD exacerbation. We assume that this information could both facilitate the hospitalization decision during the patient evaluation process and provide data to ward off exacerbation of patients with COPD, as well as arrange their outpatient treatment through the support of further clinical studies.

## 2. Materials and methods

### 2.1. Study design

Prior to this study, legal ethics approval was granted by the Pamukkale University Non-Interventional Clinical Research Ethics Committee (PAU 28.02.2020/04). This single-center, prospective study was conducted in the ED of Pamukkale University Hospital over an eight-month period (September 2020–May 2021). All consecutive subjects with the clinical manifestation of COPD exacerbation and who gave written informed consent were recruited for the study. The diagnosis of COPD was accepted based on the patients’ statements and the overlap of the information in the personal medical archives of the drugs they used. The diagnosis of exacerbation has been defined as an increase in the amount or purulence of cough and sputum with an increase in shortness of breath in patients with COPD^1^. Whereas the treatment plans were generated by the primary physicians, ultrasonography measurements were performed by the ultrasonography practitioners of the study staff. The subjects under routine long-term oxygen therapy at home still received their oxygen supply at the level used at home during the measurements.

Contact information, demographic data, body weight-height information, concomitant diseases, number of admissions to a healthcare facility in the last year due to COPD exacerbation, laboratory parameters, ultrasonographic measurements of the respiratory muscles at the bedside, and clinical outcomes were noted in the study form. The criteria for admission to the intensive care unit (ICU) were unstable vital signs and clinical deterioration, development of a need for mechanical ventilation, severe shortness of breath that is unresponsive to treatment, decrease in the level of consciousness, and no improvement in hypoxemia or respiratory acidosis despite adequate oxygen and noninvasive mechanical ventilation therapy. The criteria for discharge from the ED included regression in acute symptoms, stable vital signs, and no disturbance in laboratory findings^1^. The endpoints were considered hospitalization (ward and ICU) or discharge from the ED, mortality within 15 days, or readmission within 15 days. Readmission within 15 days was defined as presenting to health institutions due to COPD exacerbation within 15 days following the first admission. The subjects were contacted via telephone 15 days after their admission for evaluation of the clinical course and endpoints.

### 2.2. Study population

Subjects over 18 years old admitted to the ED with shortness of breath and diagnosed with COPD exacerbation were included in the study. The exclusion criteria were as follows: diaphragmatic paralysis, emergency intubation or cardiopulmonary resuscitation on admission, neuromuscular disease, lung malignancy, lung or diaphragmatic surgery, terminal stage malignancy, massive ascites in the abdomen, chest wall deformities that impede measurement, pregnancy, recurrent admissions, and refusal to give informed consent.

### 2.3. Ultrasonographic considerations

The clinical data were gathered by two practitioners who had previously received formal ultrasound training and actively performed ultrasound during patient care and management. To achieve standardization, these practitioners were provided with three h of theoretical and practical training on respiratory muscle ultrasound prior to the study.

Ultrasonographic measurements were performed with the Terason uSmartTM 3200T (Teratech Corporation, Burlington, MA, United States) using 2–5 MHz and 4–15 MHz transducers during tidal breathing, with the bedside elevated at 30°–45° and the subject seated in a semirecumbent position. Each measurement was performed twice, and the mean score of the relevant measurements was calculated for each dataset.

#### 2.3.1. Measurement of diaphragmatic excursion

**Figure 1:**
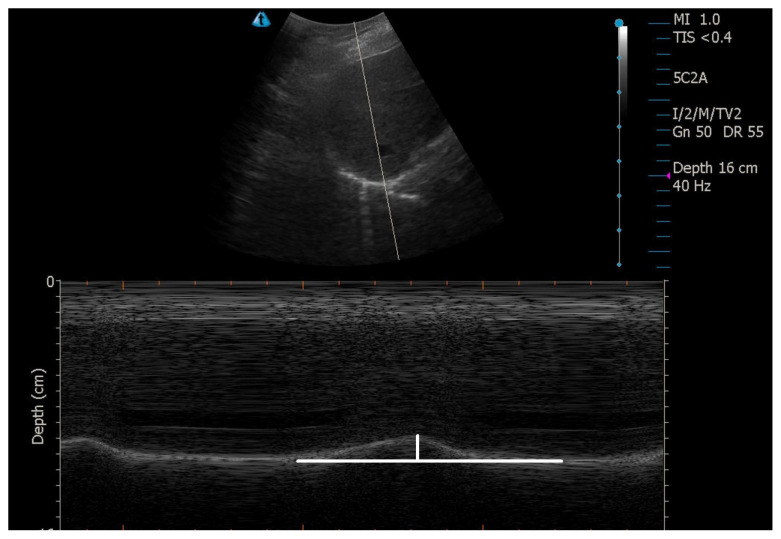
Measurement of diaphragmatic excursion.

A 2–5 MHz convex transducer was placed between the clavicular–anterior axillary line, perpendicular to the diaphragm from the subcostal area, and diaphragmatic excursion was measured using the M-mode. After capturing an image containing approximately 4–5 respiratory cycles, in which the motion of the diaphragm during inspiration and expiration can be clearly evaluated, the image was fixed, and the measurement was performed (Figure 1). The difference between the peaks of diaphragmatic motion during inspiration and expiration was measured in centimeters.

#### 2.3.2. Measurement of diaphragmatic thickness and diaphragmatic thickening fraction

**Figure 2:**
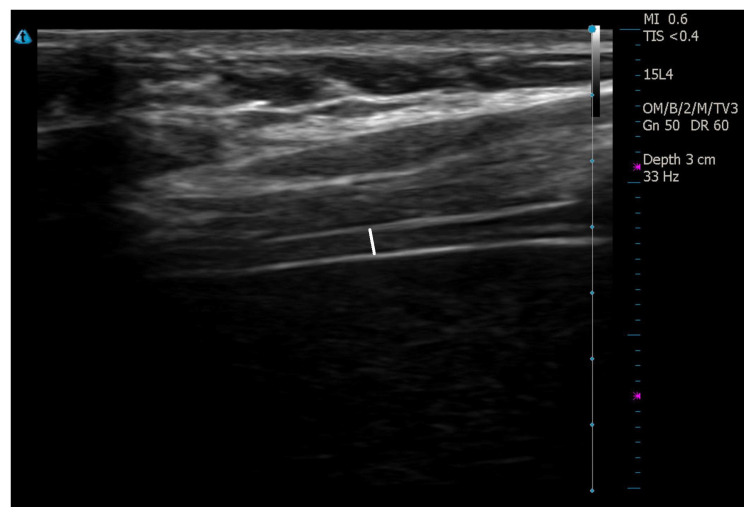
Measurement of diaphragmatic thickness.

The measurement was performed using a 4–15 MHz linear transducer from the 8^th^–9^th^ intercostal space between the anterior and mid-axillary lines. Diaphragmatic muscle thickness was measured at the end of expiration and inspiration (Figure 2). The diaphragmatic thickening fraction was calculated, based on the following formula: Diaphragmatic thickening fraction: [(end-inspiratory diaphragmatic thickness-end-expiratory diaphragmatic thickness)/end-expiratory diaphragmatic thickness] × 100.

#### 2.3.3. Measurement of intercostal muscle thickness

**Figure 3:**
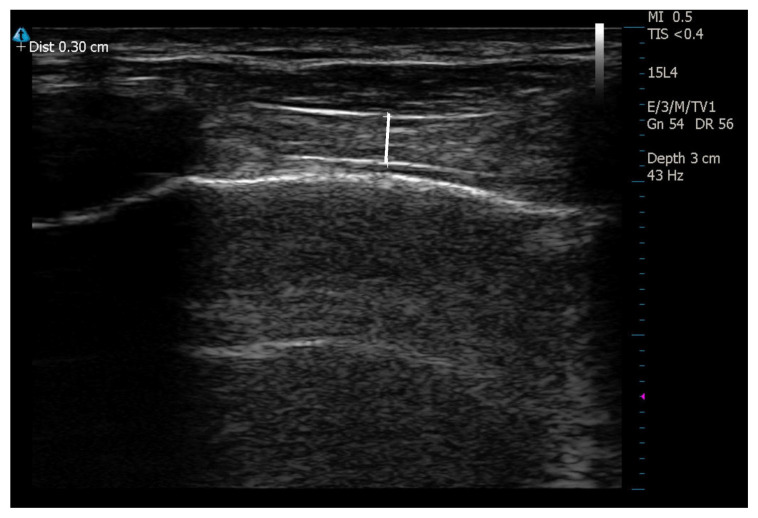
Measurement of intercostal muscles.

The measurements were performed with a 4–15 MHz linear transducer located in the sagittal plane of the parasternal region. The window was obtained from the right and left 3^rd^ intercostal space, and the measurement of the muscle was performed at the end-inspirium from the midline between the two consecutive ribs visualized (Figure 3).

### 2.4. Statistical analysis

SPSS v.25 (IBM SPSS Statistics 25 (Armonk, NY: IBM Corp.) software was used for all statistical analyses. The continuous variables were reported as mean ± standard deviation and median (minimum–maximum values). The Kolmogorov–Smirnov and Shapiro–Wilk tests were carried out to test the normality assumptions of the dataset. For independent group comparisons, an independent samples t-test and one-way analysis of variance (post hoc: Tukey) were performed in the case of parametric test assumptions. The Mann–Whitney U test and Kruskal–Wallis variance analysis (post hoc: Mann–Whitney U test with Bonferroni correction) were performed when the parametric test assumptions were violated. Categorical variables were evaluated using a chi-square test. Receiver operating characteristic (ROC) analysis was performed to determine the diagnostic performance of the variables. The Youden index value derived as a result of ROC analysis was used to identify the most appropriate cut-off point. Sensitivity and specificity values were used to evaluate the diagnostic performance of the variables. In investigating the association between nonnormally distributed variables, the correlation coefficients and their significance were calculated using the Spearman test. The correlation between the two measurements was calculated using the intraclass correlation coefficient (ICC). The statistical significance was set at p ≤ 0.05 for all the statistical analyses.

## 3. Results

### 3.1. Demographic data

**Figure 4:**
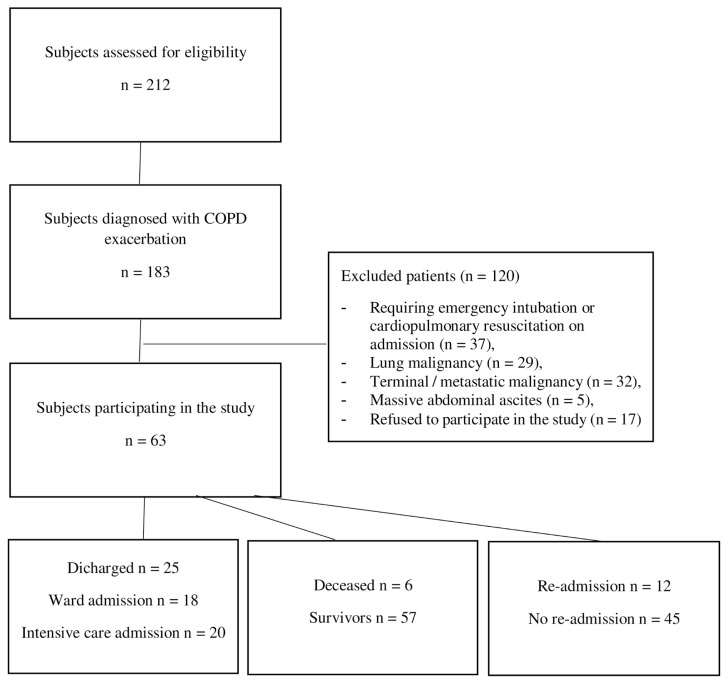
Flowchart of the study.

Of the 183 subjects presenting with COPD exacerbation, 63 were considered eligible and recruited for the study. Those refusing to give their informed consent (n = 17), requiring emergency intubation or cardiopulmonary resuscitation on admission (n = 37), or afflicted with lung malignancies (n = 29), terminal malignancies (n = 32), and massive ascites in the abdomen (n = 5) were excluded from the scope of the study (Figure 4).

There were 63 eligible subjects. Forty-four (69.8%) of them were male (mean age 70.3 ± 8.98 years; age range 56–89 years). The participating subjects had received a COPD diagnosis for an average of 15.44 ± 7.93 (min 4–max 40) years. Six (9.28%) subjects had never smoked in their lives, while the smokers had a smoking history of 25.9 pack-years on average. The most prevalent comorbid/chronic disease among the study population was diabetes mellitus, which was observed in 14 (22%) subjects (Table 1).

Of the total study population, 25 (39.7%) were discharged from the ED, 18 (28.6%) were admitted to the ward, and 20 (31.7%) were admitted to the ICU. Mortality occurred in 6 (10%) subjects. The number of readmissions within 15 days was 12 (21%).

### 3.2. Comparison of the subjects discharged from the ED or hospitalized in the ward and ICU

The mean age of the hospitalized subjects was higher than that of the discharged subjects (72.5 ± 8.82 vs. 67.1 ± 8.4 years, respectively; p = 0.023). The hospitalized subjects had lower body mass index (BMI) (p = 0.001), longer COPD duration (p = 0.007), and more frequent admission due to exacerbation in the last year (p < 0.001). When the subjects were divided into the subgroups of discharge, ward, and ICU (Table 2), the ICU subjects exhibited lower BMI, longer COPD duration, and more frequent admission in the last year due to COPD exacerbation (all p < 0.05). Further, in the ICU admission group, diaphragmatic excursion and end-expiratory diaphragmatic thickness were lower than the ward admission and discharge groups, respectively. Also, right and left intercostal muscle thicknesses were lower than the ward admission and the discharge groups, respectively. These values differed significantly among the three groups (p < 0.001) (Table 2).

### 3.3. Comparison of the cases resulting in mortality

Cases that ended in mortality within 15 days had more advanced age and more frequent admissions due to exacerbated COPD in the last year (all p < 0.05). The deceased subjects also had significantly reduced diaphragmatic excursions, end-expiratory diaphragmatic thickness, and intercostal muscle thickness in comparison to survivors (all p < 0.05) (Table 3).

### 3.4. Comparison of the subjects by readmission within 15 days

When the subjects were compared in relation to readmission due to relapse within 15 days, no significant difference was evident between diaphragmatic excursion, end-expiratory diaphragmatic thickness, and intercostal muscle thickness (all p > 0.05) (Table 4).

### 3.5. Diagnostic performance of respiratory muscle ultrasound

ROC analysis revealed that a diaphragm excursion value of 3.25 cm was significant for discriminating discharge from the ED, with 100% sensitivity and selectivity (AUC = 1). Regarding admission to the ICU, a diaphragm excursion threshold value of 1.82 cm was significant for discrimination, with 100% sensitivity and selectivity (AUC = 1). Accordingly, all subjects with diaphragmatic excursions greater than 3.25 cm were discharged (n = 25), with no subject discharged under this cut-off point. Moreover, all the subjects with a value less than 1.82 cm were hospitalized in the ICU (n = 20), with no subject above this cut-off point admitted to the ICU (Table 5).

### 3.6. The relationship between PaCO_2_ and respiratory muscles

We observed a moderate negative correlation between PaCO_2_ values and diaphragmatic excursion and end-expiratory diaphragmatic thickness (r = −0.628 and r = −0.657, respectively; p < 0.001), a moderate positive correlation between PaCO_2_ values and diaphragmatic thickening fraction (r = 0.399; p = 0.001), and a moderate negative correlation between PaCO_2_ values and right and left intercostal muscle thicknesses (r = −0.381 and r = −0.417; p < 0.01, respectively).

### 3.7. Interrater agreement between ultrasound practitioners

The ICC values for diaphragmatic excursion, end-expiratory diaphragmatic thickness, and right and left intercostal muscle thickness were calculated as 0.977, 1.000, 1.000, and 0.992, respectively (Table 6).

## 4. Discussion

Although diaphragmatic thickness can be enhanced with increased airflow resistance in COPD, diaphragmatic atrophy and reduced force-generating capacity of the diaphragm are also likely to appear owing to oxidative stress, sarcomeric injury, activated proteolytic machinery, contractile protein degradation, and systemic inflammatory features [11]. These two opposing mechanisms may account for the inconsistent values of diaphragmatic thickness reported in the literature. Moreover, the presence of these underlying mechanisms may involve subacute or chronic processes, which can explain the association between disease severity and respiratory muscle ultrasound in stable patients with COPD. According to the results of our study, diaphragmatic excursion, diaphragmatic end-expiratory thickness, and right and left intercostal muscle thicknesses were lower in ED patients with exacerbated COPD, resulting in worse outcomes.

The diaphragmatic excursion was shown to be 0.9–2.8 cm during ventilation at rest, although this range can reach as large as 3.3–9 cm during deep ventilation [18]. Due to hyperinflation, the diaphragm is located at a lower level of the xiphoid in patients developing advanced COPD compared to the normal population, reducing its operating range and impairing the inspiratory capacity of the muscles [19]. In patients with COPD, diaphragmatic excursion has been associated with parameters characterizing the severity of the disease, such as the number of admissions in the last year [20,21]. Compared with healthy controls, diaphragmatic motion in stable patients with COPD was 14.7 ± 5.5 (3.0–27.8) mm and was greater and faster than in normal subjects [18,22]. However, another study revealed significantly reduced diaphragmatic excursions in the deep inspiration of patients with COPD compared to those of the age-matched control group (45.0 ± 12.8 mm vs. 64.6 ± 6.3 mm) [23]. Although these studies were generally carried out on stable subjects under outpatient follow-up, our research addressed diaphragmatic excursions performed on subjects manifesting acute exacerbation, having a poor clinical course, or ending up with mortality. Considering the values reported in the literature, diaphragmatic excursions of our subjects in critical condition turned out to be substantially reduced compared to those of stable subjects.

There are conflicting data on the association of the diaphragmatic thickening fraction in the weaning phase of endotracheal intubation and assessing the success of noninvasive mechanical ventilation [17, 24–27]. Some lines of research tend to attribute lower values of diaphragmatic thickening fraction to weaning or noninvasive ventilation failure [24–26]. However, some of them showed positive results from ultrasonography of skeletal muscles instead of respiratory muscles [17]. A study correlating mortality and length of hospital stay concluded that a diaphragmatic thickening fraction below the critical threshold of 29% might result in increased length of ICU stay, length of stay on mechanical ventilators, ICU mortality, and in-hospital mortality [27]. We did not evaluate striated muscles in our study. We would expect the ultrasonography of the respiratory muscles of our critical patients, especially those who were admitted to the ICU or resulted in mortality, to be close to those published in the literature, which focuses mainly on ICU patients. Further, in previous studies, patients with more diverse diseases, including COPD, than our patient group was highlighted. Therefore, the differences in the findings may be attributed to disease mechanisms and the related complex mechanical and inflammatory processes.

The adaptive mechanisms and systemic inflammatory response induced by COPD act on parasternal intercostal muscles, and have been associated with age and the spirometric severity of the disease [28,29]. These findings not only confirm the inspiratory muscle atrophy observed in our COPD subjects but also lend support to the advanced age and low BMI of the ICU subjects in our study.

Decreased diaphragmatic and intercostal muscle thickness are more likely to result in prolonged mechanical ventilation and ICU stay, as well as in-hospital mortality [30]. An experimental prospective study on rabbits mechanically ventilated for 51 h reported lower diaphragm/body weight and intercostal muscle mass/body weight than the controls, in addition to alterations in the muscle fibers and their contractile properties [31]. These outcomes may shed light on the complications that develop in subjects with decreased respiratory muscle thickness. Our findings concerning intercostal muscle measurements can be attributed to the advanced age of the subjects and advanced disease severity, rather than to the complications associated with mechanical ventilation.

A primary limitation of our study is the small sample size. This is mainly due to the exclusion of other subject groups that could complicate COPD. Patients who were unstable and were immediately intubated or underwent cardiopulmonary resuscitation immediately at the time of arrival to the ED, or were already intubated at the admission to the ED were also excluded. These correspond to a high number of patients. However, a certain period of time is required for ultrasound evaluation and since diagnostic ultrasound applied in the ED cannot interfere with life-saving procedures, at the same time it may not be possible to give these patients the position determined in the methodology section, these patients have been excluded from the study. Further, the frequency of patients with COPD exacerbations decreased during the study period, given the widespread use of masks due to the COVID-19 pandemic during working hours, and the restrictions imposed especially on those with underlying chronic diseases. Another reason for the limited research population is that the patients who applied to our center were predominantly complicated cases, and the cases who could be clinically relieved with early treatment were referred to other state hospitals by the prehospital ambulance team. Further, we did not interfere with the diagnosis and patient care procedures of the primary physicians of the patients. Therefore, leukocyte counts that may indicate acute inflammation were included in the statistics, and values of other laboratory parameters, such as C-reactive protein (CRP) and procalcitonin, which were not studied in all patients, were not included in the statistics.

## 5. Conclusions

Taken together, our results suggest that diaphragmatic excursion, diaphragmatic end-expiratory thickness, and right and left intercostal muscle thickness are highly augmented in the prognosis of subjects admitted due to COPD exacerbation in emergency settings. Bedside ultrasound of these respiratory muscles proves to be a new, promising tool for predicting the outcome of patients with COPD exacerbations in the ED.

## Figures and Tables

**Table 1:** Demographic characteristics of the subjects.

Age, years		70.3 ± 8.98
Gender	Female / Male	19/44 (30.2/69.8)
Smoking, pack.years		25 ± 15.3
Body mass index		26.47 ± 6.00
COPD duration, years		15.44 ± 7.93
Number of admissions to hospital in the last one year due to COPD exacerbation		1.5 ± 1.3
Chronic diseases	Diabetes mellitus	14 (22)
	Hypertension	12 (19)
	Heart failure	8 (13)
	Chronic renal failure	7 (11)
	Coronary artery disease	6 (10)
	Benign prostatic hyperplasia	4 (6)
	Hypothyroidism	3 (5)
	Others	6 (10)

The continuous variables were shown as mean ± standard deviation (SD); the categorical variables were shown as number (%).

**Table 2:** Evaluation of respiratory muscle measurements in terms of discharge, ward admission, and ICU admission.

	Discharge, (n = 25)	Ward admission, (n = 18)	ICU admission, (n = 20)	p-value
Female, n (%)*	8 (32)	5 (28)	6 (30)	0.957
Age, years†	65 (56–80)	71 (56–85)	75 (60–89)	0.051
Respiratory rate/min†	25 (16–40)	24 (16–40)	24 (18–60)	0.795
Body mass index†	29.8 (17.6–37.2)	29.7 (23.5–36.1)	18.9 (16.4–27.6)	**< 0.001** a b
Smoking, pack.years†	20 (5–50)	25 (0–40)	27.5 (0–70)	0.234
COPD duration, years†	13 (5–35)	13 (5–20)	19 (8–40)	**0.007** a b
Number of admissions in the last one year†	1 (0–2)	1 (0–4)	2 (1–5)	**< 0.001** a b
Diaphragmatic excursion, cm‡	3.95 ± 0.39	2.53 ± 0.29	1.13 ± 0.21	**< 0.001** a b c
Diaphragmatic end-expiratory thickness, mm†	2.9 (2.65–3.2)	2.55 (2.2–3.2)	2.07 (1.86–2.3)	**< 0.001** a b c
Diaphragmatic thickening fraction, %†	49.6 (34.4– 56.8)	47.8 (25–56)	51.4 (38.1–58.1)	0.086
Intercostal muscle thickness, left, mm‡	4.57 ± 0.47	3.78 ± 0.77	3.12 ± 0.208	**< 0.001** a b c
Intercostal muscle thickness, right, mm‡	4.6 ± 0.44	3.75 ± 0.75	3.09 ± 0.25	**< 0.001** a b c
PaCO_2_, mmHg†	46 (31–50)	50.5 (36–65)	64.5 (47–80)	**< 0.001** a b
Leukocyte count,/mcL‡	9600 ± 2140	10,992 ± 1980	12,850 ± 2470	**< 0.001** a b c

a: Comparison of ward admission and ICU admission;

b: Comparison of discharge and ICU admission;

c: Comparison of discharge and ward admission;

*: Chi Square Test;

†: Kruskal Wallis Variance Analysis (median (min–max));

‡: One Way Analysis of Variance (mean ± SD);

ICU: Intensive care unit.

**Table 3:** Respiratory muscle measurements of the deceased subjects and survivors.

	Deceased subjects, (n = 6)	Survivors, (n = 57)	p-value
Female, n (%)^a^	4 (67)	15 (26)	0.062
Age, years^b^	80 ± 4.2	69.7 ± 8.7	**< 0.001**
Respiratory rate/min^c^	30 (18–60)	24 (16–40)	0.24
Body mass index^c^	22.6 (17.6–27.6)	28.6 (16.4–37.2)	0.065
Smoking, pack.years^c^	40 (0–60)	25 (0–70)	0.13
COPD duration, years^b^	23.3 ± 13.3	14.61 ± 6.8	0.17
Number of admissions in the last one year^c^	2.5 (2–4)	1 (0–5)	**0.010**
Diaphragmatic excursion, cm^c^	1.01 (0.80–1.20)	2.60 (0.87–4.70)	**< 0.001**
Diaphragmatic end-expiratory thickness, mm^b^	2.03 ± 0.12	2.58 ± 0.49	**0.008**
Diaphragmatic thickening fraction, %^c^	51.45 (38.1–52.38)	49.31 (25–58.14)	0.51
Intercostal muscle thickness, left, mm^b^	3.08 ± 0.19	3.97 ± 0.79	**< 0.001**
Intercostal muscle thickness, right, mm^b^	3.79 ± 0.19	3.90 ± 0.82	**< 0.001**
PaCO_2_, mmHg^c^	48.25 (37–67)	48.5 (31–80)	**0.02**
Leukocyte count/mcL^b^	14,300 ± 1520	10,670 ± 2410	**0.001**

a: Chi Square Test;

b: Independent Samples t-test (mean ± SD);

c: Mann Whitney U test (median (min–max));

ICU: Intensive care unit.

**Table 4:** Respiratory muscle measurements of the subjects with and without readmissions to hospital within 15 days.

	Subjects with readmissions, (n = 12)	Subjects without re-admissions, (n = 45)	P Value
Female, n (%)^a^	4 (33)	11 (24)	0.71
Age, years^b^	71.83 ± 8.79	68.71 ± 8.74	0.28
Respiratory rate/min^c^	22 (16–40)	24 (16–40)	0.18
Body mass index^c^	29.9 (19.2–35.0)	26.3 (16.4–37.2)	0.053
Smoking, pack.years^c^	25 (5–50)	25 (0–70)	0.86
COPD duration, years^c^	15 (5–25)	13 (5–35)	0.47
Number of admissions in the last year^c^	1 (0–4)	1 (0–5)	0.31
Diaphragmatic excursion, cm^c^	3.50 (1.25–4.80)	2.70 (0.70–4.70)	0.32
Diaphragmatic end-expiratory thickness, mm^b^	1.35 ± 0.17	1.37 ± 0.30	0.81
Diaphragmatic thickening fraction, %^c^	51.01 (45.45–56.55)	48.92 (25.00–58.14)	0.13
Intercostal muscle thickness, left, mm^c^	4.43 (3.10–5.10)	3.75 (2.60–5.36)	0.072
Intercostal muscle thickness, right, mm^c^	3.15 (2.80–3.30)	4.05 (2.60–5.35)	0.06
PaCO_2_, mmHg^c^	48.25 (37–67)	49 (31–80)	0.98
Leukocyte count/mcl^b^	10,660 ± 2520	10,680 ± 2410	0.98

a: Chi Square Test;

b: Independent Samples t-test (mean ± SD);

c: Mann Whitney U test (median (min–max)).

**Table 5:** Diagnostic performance of respiratory muscle ultrasound.

		Diaphragmatic excursion, cm	Diaphragmatic end-expiratory thickness, mm	Diaphragmatic thickening fraction, %	Intercostal muscle thickness, left, mm	Intercostal muscle thickness, right, mm
Discharge	AUC (%95 CI lower–upper)	1 (1–1)	0.842 (0.742–0.942)	0.498 (0.351–0.645)	0.902 (0.827–0.977)	0.919 (0.849–0.989)
	Cut-off value	3.25	1.37	49.1	3.82	3.87
	Sensitivity, (%)	100	84	68	96	96
	Specificity, (%)	100	81.6	50	79	79
ICU admission	AUC (%95 CI lower–upper)	1 (1–1)	0.981 (0.952–1)	0.647 (0.5–0.793)	0.895 (0.81–0.979)	0.904 (0.825–0.983)
	Cut-off value	1.82	1.12	48.4	3.53	3.45
	Sensitivity, (%)	100	90	80	100	100
	Specificity, (%)	100	98	46.5	83.7	86
Mortality	AUC (%95 CI lower–upper)	0.918 (0.843–0.993)	0.849 (0.719–0.98)	0.585 (0.372–0.798)	0.826 (0.722–0.93)	0.864 (0.763–0.965)
	Cut-off value	1.35	1.06	49.48	3.37	3.35
	Sensitivity, (%)	100	83	83	100	100
	Specificity, (%)	79	81	54	70	72

ICU: Intensive care unit.

**Table 6:** Comparison of inter-practitioner ultrasonographic measurements.

	Practitioner 1	Practitioner 2	P Value	ICC	%95 CI (lower–upper)
	Mean ± SD	Mean ± SD			
Diaphragmatic excursion, cm	12.51 ± 10.20	12.15 ± 9.53	0.332	0.977	0.991–0.999
Diaphragmatic end-expiratory thickness, mm	4.11 ± 5.19	4.09 ± 5.32	0.822	1	0.998–1.000
Intercostal muscle thickness, right, mm	5.74 ± 8.71	5.70 ± 8.80	0.524	1	0.999–1.000
Intercostal muscle thickness, left, mm	3.64 ± 1.36	3.62 ± 1.32	0.709	0.992	0.972–0.998

ICC: Intraclass correlation coefficient.

## Footnotes

1 Global Initiative for Chronic Obstructive Lung Disease (GOLD) (2022). Global strategy for the diagnosis, management, and prevention of chronic obstructive pulmonary disease 2022 report [online]. Website https://goldcopd.orgfile:///C:/Users/user/Downloads/GOLD-REPORT-2022-v1.1-22Nov2021_WMV%20(1).pdf [accessed 7 April 2022].
